# Effect of Collapsed Duplications on Diversity Estimates: What to Expect

**DOI:** 10.1093/gbe/evy223

**Published:** 2018-10-26

**Authors:** Diego A Hartasánchez, Marina Brasó-Vives, Jose Maria Heredia-Genestar, Marc Pybus, Arcadi Navarro

**Affiliations:** 1 Institute of Evolutionary Biology (Universitat Pompeu Fabra – CSIC), PRBB, Barcelona, Catalonia, Spain; 2 Departament de Ciències Experimentals i de la Salut, Universitat Pompeu Fabra, Barcelona, Catalonia, Spain; 3 Laboratoire de Biométrie et Biologie Évolutive UMR 5558, Université de Lyon, Université Lyon 1, CNRS, Villeurbanne, France; 4 National Institute for Bioinformatics (INB), Barcelona, Catalonia, Spain; 5 Centre for Genomic Regulation (CRG), Barcelona, Catalonia, Spain

**Keywords:** copy number variation, neutrality statistics, interlocus gene conversion

## Abstract

The study of segmental duplications (SDs) and copy-number variants (CNVs) is of great importance in the fields of genomics and evolution. However, SDs and CNVs are usually excluded from genome-wide scans for natural selection. Because of high identity between copies, SDs and CNVs that are not included in reference genomes are prone to be collapsed—that is, mistakenly aligned to the same region—when aligning sequence data from single individuals to the reference. Such collapsed duplications are additionally challenging because concerted evolution between duplications alters their site frequency spectrum and linkage disequilibrium patterns. To investigate the potential effect of collapsed duplications upon natural selection scans we obtained expectations for four summary statistics from simulations of duplications evolving under a range of interlocus gene conversion and crossover rates. We confirm that summary statistics traditionally used to detect the action of natural selection on DNA sequences cannot be applied to SDs and CNVs since in some cases values for known duplications mimic selective signatures. As a proof of concept of the pervasiveness of collapsed duplications, we analyzed data from the 1,000 Genomes Project. We find that, within regions identified as variable in copy number, diversity between individuals with the duplication is consistently higher than between individuals without the duplication. Furthermore, the frequency of single nucleotide variants (SNVs) deviating from Hardy–Weinberg Equilibrium is higher in individuals with the duplication, which strongly suggests that higher diversity is a consequence of collapsed duplications and incorrect evaluation of SNVs within these CNV regions.

## Introduction

Segmental duplications (SDs) are defined as ≥1 kb blocks of DNA that are present at several sites in the genome and that present ≥90% sequence similarity between copies (Sharp et al. 2006). They are an ubiquitous characteristic of eukaryotic genomes presenting complex patterns of evolution. First, SDs undergo interlocus gene conversion (IGC), also referred to as nonallelic or ectopic gene conversion, which drives their concerted molecular evolution (Walsh, 1987; Chen et al. 2007) and is a source of variation (Innan, 2002); second, genes residing within SDs may suffer different selective pressures giving rise to subfunctionalization or neofunctionalization (He and Zhang, 2005; Teshima and Innan, 2008; Assis and Bachtrog, 2013); and, third, SDs are mediators of nonallelic homologous recombination (NAHR), a common source of further duplications and deletions that can give rise to copy number variants (CNVs), which are regions of the genome that are variable in copy number in the population (Conrad and Hurles, 2007), and which are in turn associated with susceptibility to disease (Stankiewicz and Lupski, 2010).

The detection and characterization of SDs and CNVs is of great importance in the field of genomics. However, when constructing genome assemblies, duplications are prone to be collapsed into one single region due to high identity between duplicates. Collapsed duplications are known to be a particularly widespread problem of reference genomes constructed by means of whole genome shotgun assembly (Salzberg and Yorke, 2005; Kelley and Salzberg, 2010), particularly in the case of low coverage genomes. Second generation sequencing is particularly prone to this problem, given the short length of the reads it produces (Alkan et al. 2011; Ribeiro et al. 2015). Algorithms based on depth of coverage have been developed to help solve this issue (Bailey et al. 2002; Yoon et al. 2009). These algorithms align reads to a reference genome and identify regions that have more reads aligned to them than expected for single-copy regions. An increased depth of coverage in a region of the genome that has not been previously identified as being duplicated will imply that a CNV is present in the sequenced individual and/or that this region is an unresolved duplication in the reference sequence. Some reference genomes have improved considerably thanks to these tools.

In high quality genomes, such as the human reference, it is likely that the vast majority of common SDs and CNVs has already been detected and annotated. However, for many CNVs, the reference does not include the duplicated sequence elsewhere in the genome (Kidd et al. 2010; Sudmant et al. 2015). Therefore, even for these almost-finished genomes, when short-read sequence data from a single individual are aligned to the reference, there will be some collapsed sequences. If the duplications are not in the reference, apparently stringent strategies to avoid collapsing duplications, such as not considering any read that maps to more than one location in the reference genome, will be useless. In these cases, algorithms based on depth of coverage might be more adequate, but they will have little power if coverage is low.

In any case, given that SDs are privileged targets of natural selection (Bailey and Eichler, 2006; Marques-Bonet et al. 2009; Lorente-Galdos et al. 2013); given the increasing availability of databases identifying SDs and CNVs (Sudmant et al. 2015); and given the existence of methods to avoid collapsing duplications, it is worthy to assess the action of selection within SDs and CNVs. However, most genome-wide scans for natural selection concentrate their efforts on filtering-out SDs and CNVs from their analyses in order to avoid spurious signals coming, not only from collapsed duplications, but from perfectly identified SDs (e.g., Chen et al. 2009; Enard et al. 2014).

The reason for this exclusion is that summary statistics traditionally used to detect the action of natural selection on DNA sequences have been devised with single-copy sequences in mind and cannot be applied to SDs since the latter undergo concerted evolution. This nonindependent evolution of duplicates strongly alters their site frequency spectrum and linkage disequilibrium patterns and these alterations occur even under strict neutral evolution (Teshima and Innan, 2004; Thornton, 2007; Hartasánchez et al. 2014). The main mechanism behind this concerted evolution is IGC, a copy-paste process in which variants are exchanged between duplicates. Theoretical results have shown that increased diversity within duplicates can occur as a consequence IGC between paralogous copies (Ohta, 1982; Innan 2002; Teshima and Innan, 2012; Hartasánchez et al. 2014). Basically, duplicates provide more opportunity for point mutations to arise. These mutations can then be repeatedly transferred between duplicates through IGC, increasing their diversity relative to single-copy regions. Additionally, IGC breaks up short-range linkage disequilibrium (Hartasánchez et al. 2014). As a consequence of these alterations, the distributions of neutrality statistics differ between duplications and single-copy regions (Innan, 2003; Thornton, 2007) and, thus, these statistics cannot be correctly applied to duplicated regions. The presence of duplicated regions (collapsed or not) are therefore recognized as strong confounding factors in genome scans for positive (Mallick et al. 2009) and balancing selection (Fijarczyk and Babik, 2015). To the best of our knowledge, however, there has been no systematic assessment of the expected outcome of standard statistical tests for natural selection if they are applied to duplicated regions. Beyond confirming that standard neutrality statistics cannot readily be applied to duplicated regions, we set out to describe how different IGC rates among duplications and crossover (CO) rates between them influence the outcome of these tests.

## Materials and Methods

All simulations involving duplicated regions were done with a slightly modified version of SeDuS (Hartasánchez et al. 2016). SeDuS simulates the evolution of two-copy duplicates evolving under concerted evolution and a single-copy control region. We chose a range of IGC and of CO rates between duplicates to show that summary statistics are highly dependent on these parameters: IGC = 0.5 (low), IGC = 1 (intermediate), and IGC = 5 (high); CO = 1 (low), CO = 10 (intermediate), and CO = 100 (high). For every combination of these IGC and CO rates, a set of 1,000 simulations were run with a population size of *N* = 1,000, a region length of *B* = 5,000 bp, for *t* = 100,000 generations (enough for the population to reach mutation-drift equilibrium) and a sample size of *z* = 50. CO was limited to the single-copy region and no sequence similarity restriction was imposed on IGC. The rest of SeDuS parameters were the default parameters as shown in the online tutorial (http://www.biologiaevolutiva.org/sedus/; Last accessed October 22, 2018).

SeDuS produces an output in ms format as introduced by Hudson (2002), reporting positions of segregating sites and indicating the presence of the derived or ancestral allele at every position for a sample of the population at the end of every simulation run. The SeDuS code was slightly modified in order to output data not only for the original, single-copy and duplicated blocks, but also from a collapsed sample (taking half the sample from the original block and the other half from the duplicated block).

Additionally to the results from SeDuS, we have run simulations with MSMS (Ewing and Hermisson, 2010). Simulated scenarios involve neutrality, a complete selective sweep (-SAA 40 -SaA 0), an incomplete selective sweep (-SAA 40 -SaA 20) and a case of balancing selection (-SAA 0 -SaA 40).

To calculate our set of summary statistics we used the following software packages: Evolboosting (Lin et al. 2011) to calculate *π* (Nei and Li, 1979), PopGenome (Pfeifer et al. 2014) to calculate Tajima’s *D* (Tajima, 1989) and Fay and Wu’s *H* (Fay and Wu, 2000), and SSCosi (Ramírez-Soriano et al. 2008) to calculate dh (Nei, 1987). Results for additional summary statistics are found in the Supplementary Material (supplementary table S1 and figs. S1–S3, Supplementary Material online).

Regarding the selection of flanking regions adjacent to each CNV region, in order to avoid confounding factors associated with inadequate determination of start and end positions of each CNV region, we selected flanking regions of the same size of the corresponding CNV starting 1 kb apart from the start and end annotated positions. Additionally, since it is often the case to have several annotated CNVs in close vicinity to each other, SNVs within the CNV flanking regions that overlapped with other CNVs were not considered in the calculation of *π*. Regions with <2 SNVs in either the flanking or CNV regions were excluded from the analysis.

## Simulations

To obtain neutral estimates of diversity present in duplicated regions, we ran the forward-time simulator of SDs, SeDuS (Hartasánchez et al. 2016), under a range of IGC and CO rates (see Materials and Methods). We then computed a set of four standard summary statistics (average pairwise differences, Tajima’s *D*, Fay and Wu’s *H*, and Nei’s dh, see Materials and Methods) from the data generated by SeDuS (even if we focus on these four tests, a total of 10 neutrality statistics were computed and full results are shown as Supplementary Material). Each statistic is more or less sensitive to different deviations from neutrality (e.g., more sensitivity to intermediate frequency variants) and they present varying degrees of robustness in the face of potential confounding factors such as population bottlenecks or expansions.

We compare the results of these tests between single-copy regions and duplications. The average neutral variation levels of single-copy regions are in general not strongly affected by differences in CO rates and, of course, not affected by IGC. Duplications are analyzed in two ways: What we denote as *Duplicated* are statistics applied exclusively to one of the duplicated copies. For this to happen with real data, paralogs would need to be perfectly identified and differentiated. The second case, which we denote as *Collapsed*, refers to the cases in which both paralogs are analyzed as if they were only one copy. To measure the effect of collapsed duplications, we proceed with an approximation of real-life mapping that would take half of the sequences from each paralog, which increases intermediate frequency variants, in particular for low IGC rates.

**Fig. 1.**
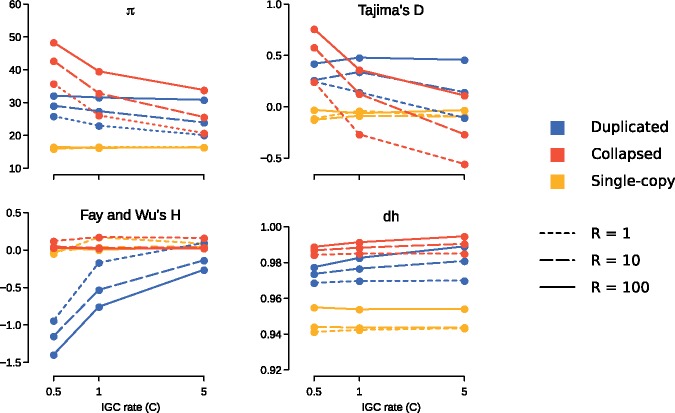
—Average values across 1,000 SeDuS simulations for average pairwise differences (*π*), Tajima’s *D*, Fay and Wu’s *H*, and Nei’s haplotype diversity (dh). Values are shown for single-copy, duplicated and collapsed for a range of CO rates (*R* = 1, 10, 100) and IGC rates (*C* = 0.5, 1, 5).

We have compared mean values from 1,000 simulation runs for each statistic for a range of IGC and CO rates (fig. 1). We confirm the reports by Innan (2003) and Thornton (2007) that tests statistics based on the standard coalescent applied to known duplications (shown in blue in fig. 1) cannot be interpreted with the same criteria as that used for single-copy regions (yellow). Furthermore, we show that values for duplicated regions vary considerably between different IGC and CO rates for some tests statistics, such as Tajima's *D*, Fay and Wu’s *H*. In the case of collapsed duplications (red), we observe a considerable overlap with values for single-copy regions for Fay and Wu’s *H*. However, nucleotide diversity (average pairwise differences, *π*) as well as the haplotype diversity estimator (dh) show little overlap and seem to be less dependent on IGC rates. In principle, therefore, these two estimators could be used to detect collapsed duplications even though there can be multiple other evolutionary histories for a single-copy region that can produce this type of signal.

**Fig. 2.**
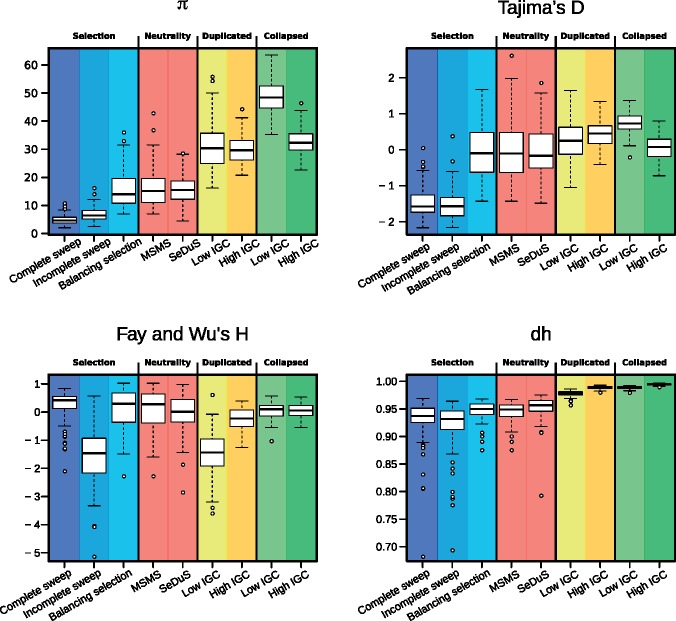
—Boxplot comparison between simulation results from MSMS (complete sweep, incomplete sweep, balancing selection and neutrality) and SeDuS (single-copy, duplicated, collapsed) with low (*C* = 0.5) and high (*C* = 5) IGC rates and CO rate *R* = 10. The length of the boxplot whiskers are 1.5 times the inter-quantile range. Distributions for Fay and Wu's *H* for an incomplete sweep resemble those from duplicates with low IGC rate. *π* and dh are two statistics that clearly differentiate between duplicates and collapsed regions from regions under selection.

Previous theoretical and simulation results have shown that elevated nucleotide diversity is achieved under low IGC and high CO rates (Hartasánchez et al, 2014). By applying our set of summary statistics we observe that this corresponds to an increase of average pairwise differences with respect to segregating sites (positive Tajima’s *D*), and an excess of high frequency derived variants (negative Fay and Wu’s *H*). Additionally, since IGC tract lengths are typically shorter than the duplicated regions, IGC events break up linkage blocks and generate a high haplotype diversity (dh). In order to test if these values are attainable under simple selective scenarios we ran coalescent simulations of single-copy regions using the MSMS software (Ewing and Hermisson, 2010) under four different models: Hard sweep, soft sweep, balancing selection and neutrality (see Materials and Methods). In figure 2, we compare simulated data for single-copy regions under selective and neutral scenarios from MSMS and simulated data for single-copy, duplicated and collapsed regions from SeDuS. Our results show, first, that when taken individually, most statistics cannot clearly distinguish selective from neutral scenarios, which is consistent with previous reports (Pybus et al. 2015); second, that in some cases, values for known duplications mimic selective signatures, such as those characteristic of incomplete sweeps in the case of Fay and Wu's *H*; and, third, that both known and collapsed duplications, given their high levels of nucleotide and haplotype diversity, could be potentially differentiated from single-copy regions or regions under selective pressures by focusing on nucleotide and/or haplotype diversity estimators.

## Collapsed Duplications in the 1,000 Genomes Project

Our results show that both, IGC between duplications and collapsed duplications, might generate an increase in measurements of diversity (average pairwise differences, for instance). We argue that due to the pervasiveness of copy-number variation in eukaryotic genomes and the difficulties of CNV detection, diversity might be consistently overestimated in CNV prone regions. Furthermore, we suggest that even within regions of the genome known to be duplicated, diversity might be overestimated due to collapsed duplications.

As a proof of concept we analyzed data from the 1,000 Genomes Project or 1 kGP (The 1000 Genomes Project Consortium, 2015). This data set has several characteristics that make it an interesting choice: First, it has been widely used since the publication of its first phase in 2010; second, it has large sample sizes from diverse human populations; third, it provides information for single-nucleotide variation and for copy-number variation (Sudmant et al. 2015); fourth, the community considers it to be a very reliable data set for estimates of single-nucleotide variant (SNV) diversity; fifth, since it is a human panel, structural variation is very well studied and resolved and therefore, not expected to cause important biases; and sixth, and crucial to our purpose, sequencing has been performed at low coverage with short-read technology.

This last point is fundamental because short-read sequencing is more prone to collapsed duplications than long-read technology. Since the latter is increasingly used, the issues caused by collapsed duplications will eventually be solved. However, large-scale sequencing efforts, such as the 100,000 Genomes Project (Turnbull et al. 2018) are still being carried-out with short-read technology, which indeed will most likely dominate diversity estimations in humans for some time to come. The same is the case for large sequencing efforts in other branches of the tree of life. Thus, the potential biases in diversity estimations caused by collapsed duplications are an important issue that should be addressed adequately.

The 1 kGP includes information not only for SNVs but also for CNVs. CNV regions were determined using an ensemble of nine different algorithms (The 1000 Genomes Project Consortium, 2015) and are annotated giving their start and end position, as well as the number of copies present in each haploid chromosome of each sample: CN0 indicates a deletion, CN1 indicates no duplication or deletion (the reference copy-number), and CN2, CN3, CN4, etc., indicate multiple number of copies, within each chromosome. We retrieved information for three populations: CEU (CEPH Utah residents with ancestry from northern and western Europe), CHB (Han Chinese in Beijing, China), and YRI (Yoruba in Ibadan, Nigeria). For each CNV region we classified each sample of our selected populations as *CN–*, *CNr*, or *CN+*, with the following criteria: CN– are samples carrying a deletion in either or both chromosomes (i.e., homozygous CN0/CN0 or heterozygous CN0/CN1), CNr are samples having the reference single-copy status on both chromosomes (i.e., homozygous CN1/CN1), and CN+ are samples carrying at least one duplication in at least one chromosome (i.e., CN2/CN2, CN2/CN3, CN3/CN3, CN1/CN2, CN0/CN2, etc.). Importantly, there are SNVs annotated within each CNV region and every sample has two annotated variants for each SNV (one from each chromosome).

The question is, then, how exactly are SNVs called within CNVs? In order to call CNVs involving duplications, reads coming from multiple copies in CN+ samples must have been collapsed onto one single region in the reference. Are these collapsed reads used to call SNVs or are have they been filtered out in some way? To answer these questions focusing on common CNVs, we performed a series of analysis independently for CEU, CHB and YRI. We selected those CNV regions for which there were at least 20 CNr individuals and at least 20 CN+ individuals, within the close to 100 samples available from each population. For each CNV region within this subset, we randomly selected a sample of 20 CNr individuals and 20 CN+ individuals and calculated the average pairwise differences (*π*) separately for each group. For the same groups defined by their copy-number status, and to ensure comparison with similar single-copy regions, we additionally calculated *π* for the single-copy 5′ and 3′ flanking regions adjacent to each CNV (see Materials and Methods).

**Fig. 3.**
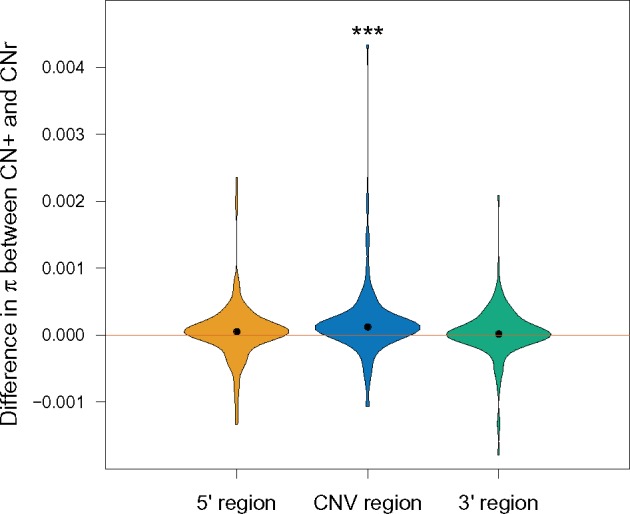
—Violin plots show the distribution of differences in average pairwise differences, *π*, between CNr and CN+ groups (CN+ minus CNr) for the CNV region (blue), and for the 5′ (yellow) and 3′ (green) regions flanking each CNV, pooling data from all CNVs and from the three populations analyzed. Black points indicate the median from each distribution. Mean increases are 15.5%, 4.7%, and 3.2%, with paired *t*-test *P*-values (represented by asterisks) of 0.181, 1.7*e*–04, and 0.326, for the 5′, CNV, and 3′ regions, respectively.

We calculated the difference in *π* between the CNr and CN+ groups (fig. 3 and supplementary table S2, Supplementary Material online). The value of *π* within the CNV region for the CN+ group is consistently higher than *π* for the CNr group (paired *t* test *P*-value of 1.4*e*–04), with a mean difference of 1.7*e*–04, which corresponds to a mean 15.5% increase of *π* within the CN+ group with respect to the CNr group. As to flanking regions, both 5′ and 3′ regions presented an increase of *π* in CN+ relative to CNr individuals, with a mean 4.7% increase within the 5′ flanking region and a 3.2% in the 3′ region, albeit with nonsignificant *P*-values. When analyzing each population separately we also observe a statistically significant difference in *π* between the CNr and CN+ groups within the CNV region in all three cases (supplementary fig. S4, Supplementary Material online).

The most plausible explanation for the higher diversity found within the CN+ group relative to the CNr group is that SNVs are called using collapsed duplications (which might or not involve IGC between copies). In principle, however, historical IGC between duplications can also cause an increase in diversity and therefore we could observe this effect even if reads coming from duplicates are being filtered out prior to SNV calling.

To provide further evidence that this increase in variation is caused by collapsed duplications (with or without IGC) we tested if SNVs within CNV regions are under Hardy–Weinberg Equilibrium (HWE). If different copies have, for example, fixed differences between them, when these positions are collapsed they will appear as heterozygous variants in all CN+ samples, and thus, clearly, not under HWE. We calculated the fraction of SNVs within each CNV which failed a Hardy–Weinberg Equilibrium test with a *P*-value smaller than 0.05, for the CNr and CN+ groups independently (supplementary fig. S5, Supplementary Material online). We find that for the three populations that we analyzed, this fraction is higher for the CN+ than for the CNr group with strong statistical significance (*P*-values for CEU, CHB, and YRI were 8.46*e*–5, 7.39*e*–10, and 5.42*e*–5, respectively). For the CNr group, many of the CNVs had all SNVs under HWE. Repeating the test excluding these CNVs we still obtain significant *P*-values (1.4*e*–2, 1*e*–4, 9*e*–4, for CEU, CHB, and YRI, respectively).

We then verified that the higher values of diversity that we had observed in the CN+ group were in fact caused by those SNVs that were not under HWE. To do so, we calculated *π* only taking into account the SNVs under HWE. The differences in *π* between the CN+ and the CNr groups became nonsignificant when considering only these variants (in fact, *π* became lower in the CN+ group, most likely containing reads from collapsed duplications). Potentially, therefore, even if we observe an overestimation of variation due to collapsed duplications in raw data such as the 1kGP, diversity estimates could be corrected by applying strict filtering criteria.

These results indicate that diversity estimations in regions identified as CNVs might be biased, showing higher diversity with respect to the real diversity present at that particular locus. Since it is known that duplications can alter diversity estimations, most genome-wide analysis remove tracts identified as duplicated or copy-number variable. The most commonly used filters to eliminate repetitive regions from analyses in humans are the repeat tracts from the UCSC Genome Browser. There are three tracts that indicate start and end positions of repeats: RepeatMasker (50% of the genome), SegDups (5%), and SimpleRepeats. Even though we expected our selected set of CNV regions to have a some overlap with the SegDups tract, which includes resolved SDs in the human reference genome, we observe that most of the CNV regions within our set do not overlap with the SegDups tract (results not shown).

## Conclusions

In this study, we show that both IGC and collapsed duplications alter the site-frequency spectrum and linkage disequilibrium patterns of duplications in such a way that standard neutrality tests cannot be applied. Furthermore, we have demonstrated that known or collapsed duplications can imitate signals of selection, emphasizing that test statistics should not be used in isolation as a means to detect natural selection. We present evidence that low-coverage human data may contain SNV calls from collapsed duplications within regions identified as copy-number variable. Diversity among individuals carrying duplications is higher than diversity among individuals not carrying the duplication with strong statistical significance in all studied populations. Furthermore, individuals carrying the duplication contain a significantly higher fraction of SNVs not under Hardy–Weinberg Equilibrium. We suggest that collapsed duplications might be a pervasive characteristic of low-coverage genomes and that they might have important consequences in diversity estimates if not taken into account.

## Supplementary Material

Supplementary Data
