# Supplementary material for: Effect of Collapsed Duplications on Diversity Estimates: What to Expect
Source: Genome Biol Evol. 2018 Oct 26;10(11):2899–905. doi: 10.1093/gbe/evy223 (PMC6239678; doi:10.1093/gbe/evy223)
Supplement: Supplementary Data [file evy223_supp.pdf]

Supplementary Material

Effect of collapsed duplications on diversity estimates: what to expect

Diego A. Hartasánchez, Marina Brasó-Vives, Jose Maria Heredia-Genestar, Marc Pybus, Arcadi Navarro

Table S1. Selected set of summary statistics applied to our data.

Test statistic	Type	Reference	Package
π	Diversity estimator	Nei and Li, 1979	Evolboosting
Watterson's θ	Diversity estimator	Watterson, 1975	Evolboosting
Tajima's D	Neutrality statistic	Tajima, 1989	PopGenome
Fu and Li's D	Neutrality statistic	Fu and Li, 1993	PopGenome
Fu and Li's F	Neutrality statistic	Fu and Li, 1993	PopGenome
Fay and Wu's H	Neutrality statistic	Fay and Wu, 2000	PopGenome
Zeng's E	Neutrality statistic	Zeng et al., 2006	PopGenome
Li's MFDM	Neutrality statistic	Li, 2011	Evolboosting
dh	Haplotype-based	Nei, 1987	SScosi
iHS	Haplotype-based	Voight *et al*., 2006	rehh

To calculate these statistics we used four programs: PopGenome (Pfeifer et al., 2014), Evolboosting (Lin et al., 2011), SSCosi (Ramírez-Soriano et al., 2008), and rehh (Gautier and Vitalis, 2011). Some of the statistics are implemented in several programs and results are largely reproducible between different software.

Table S2.

Population	Region	Number	Av. length	Group	Av. num. SNVs	Av. pi
All	All	153	18977.16		83.09	0.00106
	5'		17843.63			
				CNr	73.70	0.00108
				CN+	77.02	0.00127
	CNV		21330.66			
				CNr	99.50	0.00097
				CN+	110.10	0.00108
	3'		17757.18			
				CNr	67.67	0.00080
				CN+	70.56	0.00137
CEU	All	51	17501.38		72.98	0.00108
	5´		16343.67			
				CNr	64.67	0.00102
				CN+	71.74	0.00118
	CNV		18962.03			
				CNr	77.64	0.00103
				CN+	96.67	0.00132
	3'		17198.46			
				CNr	59.59	0.00091
				CN+	67.56	0.00102
CHB	All	63	20744.16		61.85	0.00080
	5´		18568.3			
				CNr	54.92	0.00081
				CN+	57.30	0.00086
	CNV		24218.13			
				CNr	71.89	0.00080
				CN+	77.87	0.00092
	3'		19446.05			
				CNr	54.03	0.00070
				CN+	55.11	0.00073
YRI	All	39	17922.92		117.06	0.00137
	5´		18095.47			
				CNr	103.80	0.00129
				CN+	105.41	0.00127
	CNV		19575.1			
				CNr	150.31	0.00151
				CN+	160.18	0.00166
	3'		16098.2			
				CNr	90.69	0.00126
				CN+	91.94	0.00124

Figure S1. Average values for our complete set of summary statistics from 1,000 SeDuS simulations. Values are shown for single-copy, duplicated and collapsed for a range of crossover rates (R = 1, 10, 100) and IGC rates (C = 0.5, 1, 5).

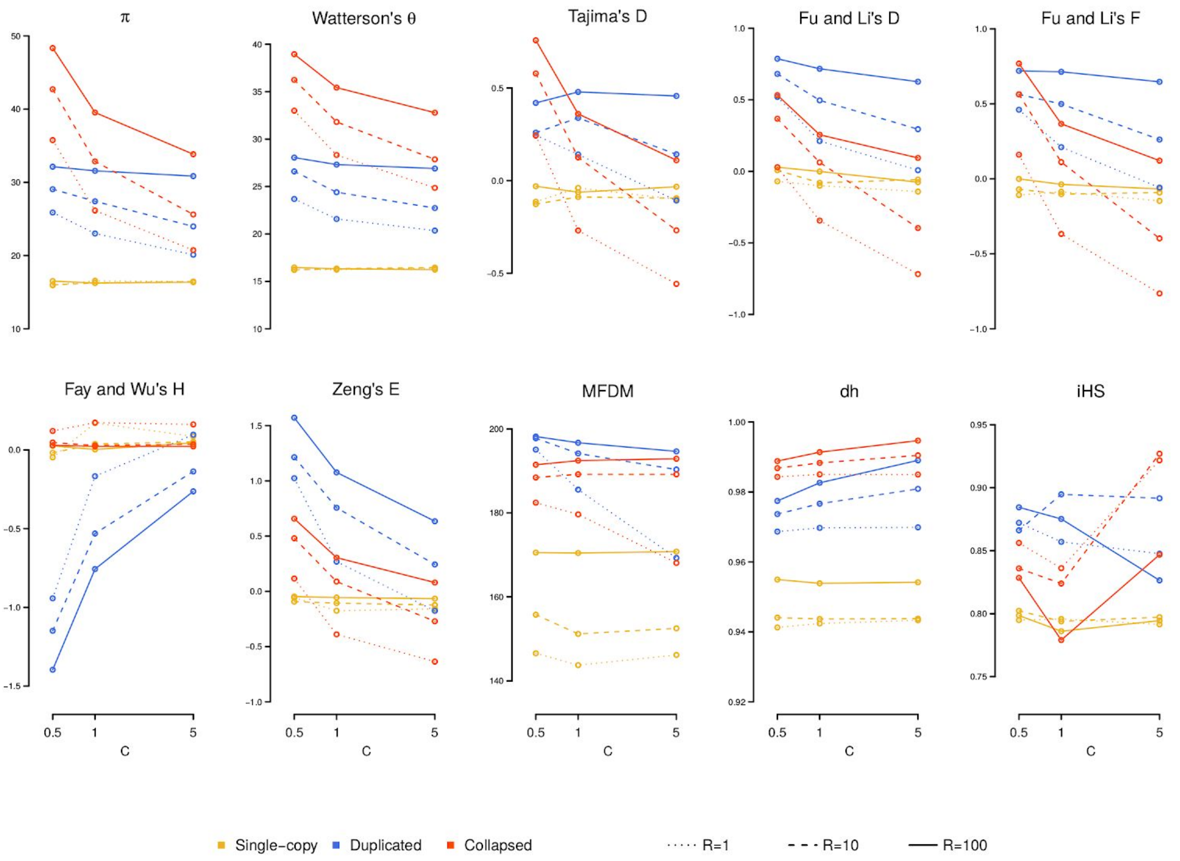

Figure S2. Distribution of values for our complete set of summary statistics from 1000 SeDuS simulations. Density plots are shown for single-copy, duplicated and collapsed for a crossover rate R = 10 and a range of IGC rates (C = 0.5, 1, 5).

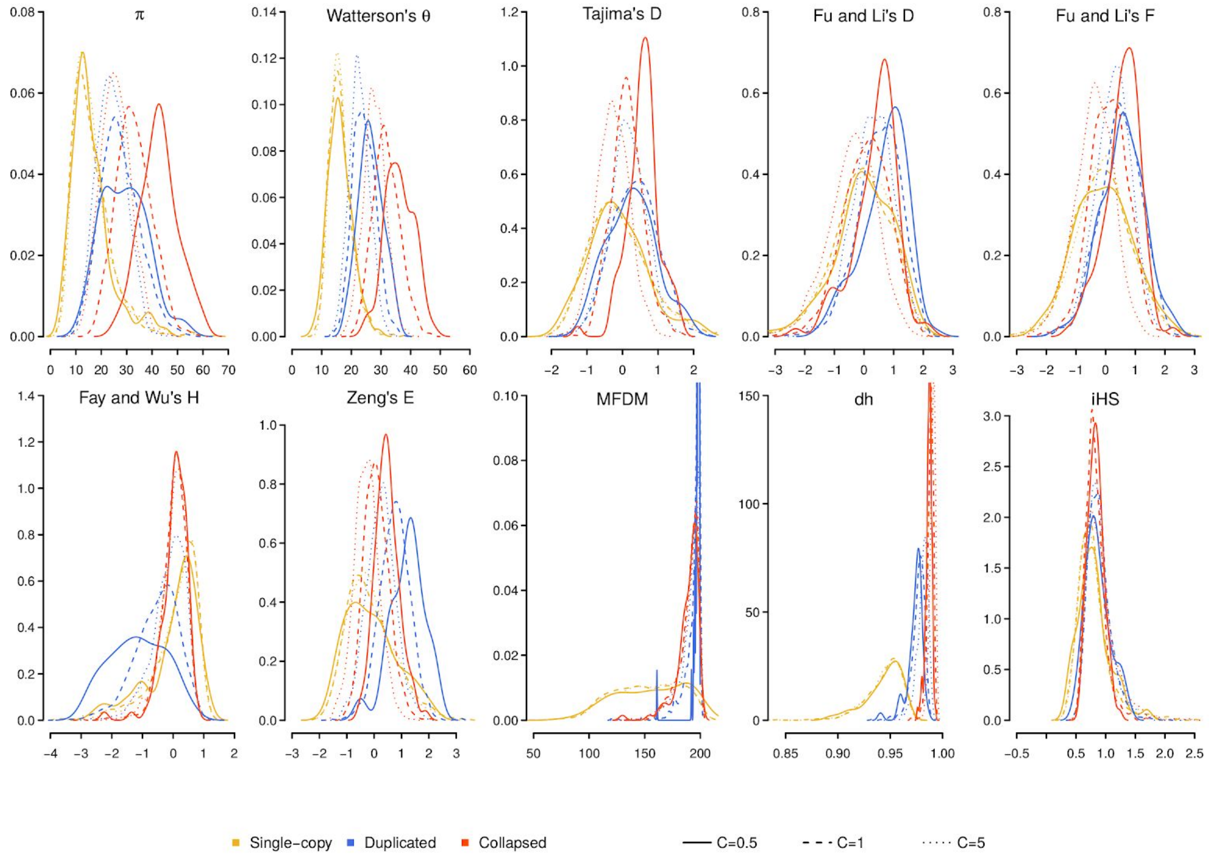

Figure S3. Boxplot comparison between simulation results from MSMS (complete sweep, incomplete sweep, balancing selection and neutrality) and SeDuS (single-copy, duplicated, collapsed) with low (C = 0.5) and high (C = 5) values of IGC rates and CO rate of R = 10 for our complete set of summary statistics. The length of the boxplot whiskers are 1.5 times the inter-quantile range.

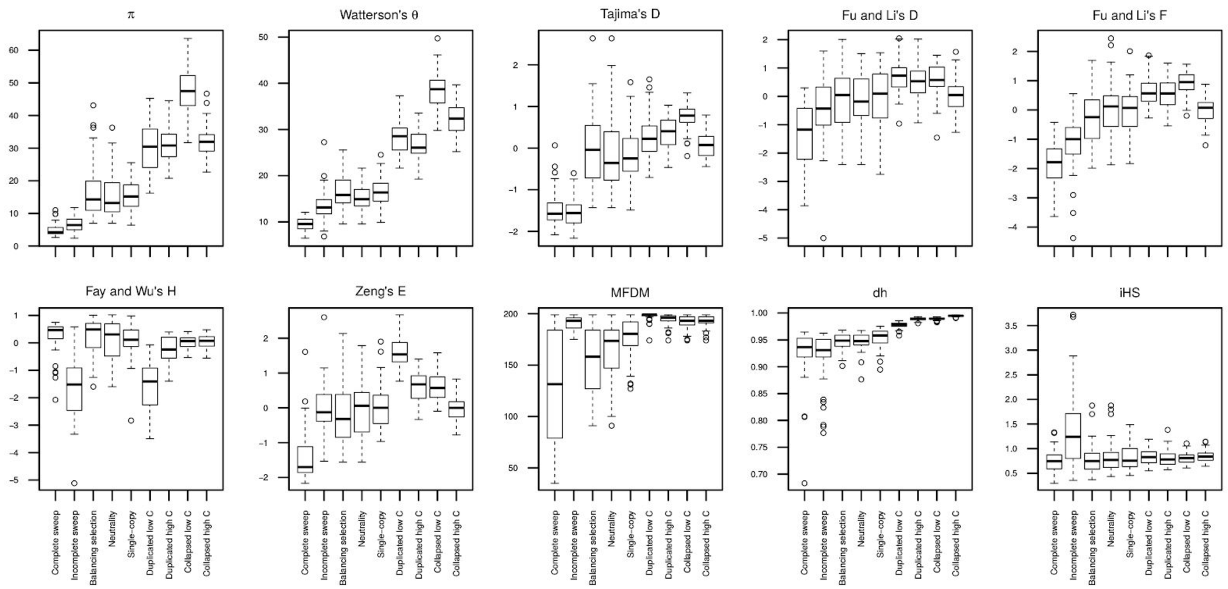

Figure S4. Violin plots show the distribution of differences in average pairwise differences, π, between CNr and CN+ groups (CN+ minus CNr) for the CNV region (blue), and for the 5’ (yellow) and 3’ (green) regions flanking each CNV, for CEU (top), CHB (middle) and YRI (bottom). Black points indicate the median from each distribution. Mean increases for the CNV region are 27.6% (CEU), 14.4% (CHB), and 9.9% (YRI) with paired t-test p-values (represented by asterisks) of 0.038, 0.014, and 0.015, respectively. Differences for the 5’ and 3’ regions are non significant for all three populations.

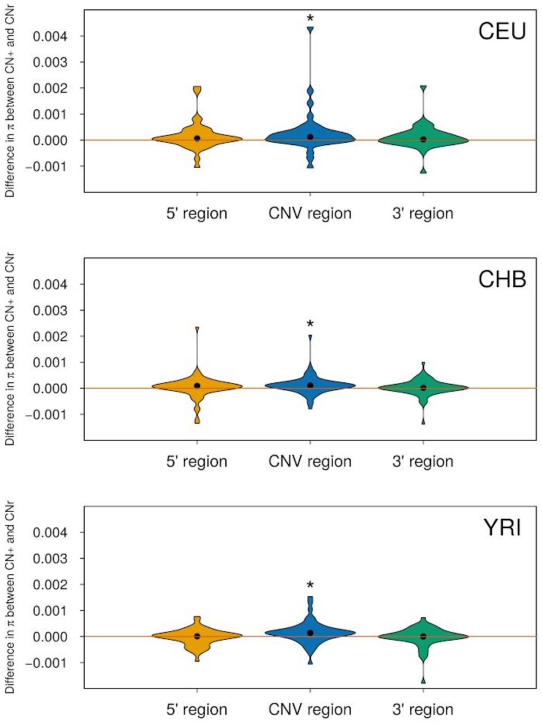

Figure S5. Fraction of SNVs within each CNV that fail a Hardy-Weinberg Equilibrium test with a p-value smaller than 0.05, for the CNr and CN+ groups independently, for CEU (left), CHB (middle), and YRI (right). This fraction is higher for the CN+ than for the CNr group with strong statistical significance for all populations. Paired t test p-values were 8.46e-5, 7.39e-10, and 5.42e-5 for CEU, CHB, and YRI respectively.

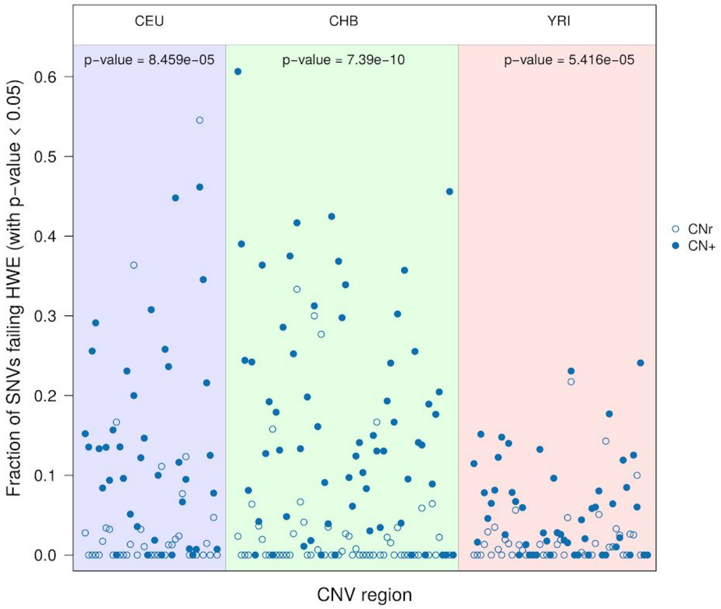

Additional references

Fu YX, Li WH. 1993. Statistical tests of neutrality of mutations. Genetics 133:693–709.

Gautier M, Vitalis R. 2012. rehh: an R package to detect footprints of selection in genome-wide SNP data from haplotype structure. Bioinformatics 28:1176-1177.

Li H. 1993. A new test for detecting recent positive selection that is free from the confounding impacts of demography. Mol. Biol. Evol. 28:365-375.

Voight BF, Kudaravalli S, Wen X, Pritchard JK. 2006. A map of recent positive selection in the human genome. PloS Biol. 4:e72.

Watterson GA. 1975. On the number of segregating sites in genetical models without recombination. Theor. Popul. Biol. 7 256–276.

Zeng K, Fu Y, Shi S, Wu C. 2006. Statistical tests for detecting positive selection by utilizing high-frequency variants. Genetics 174:1431-1439.
